# The phase composition, morphology and compressibility of graphene–zirconia composite nanostructured powder

**DOI:** 10.1039/c9na00624a

**Published:** 2019-12-17

**Authors:** E. A. Trusova, D. D. Titov, A. N. Kirichenko, E. V. Shelekhov, N. S. Trutnev, A. M. Afzal, I. A. Perezhogin

**Affiliations:** Baikov Institute of Metallurgy and Materials Science RAS, 49 Leninsky pr. Moscow 119334 Russia trusova03@gmail.com; Technological Institute for Superhard and Novel Carbon Materials 7a Tsentralnaya street, Troitsk Moscow 142190 Russia; National University of Science and Technology (MISiS) 4 Leninskiy pr. Moscow 119049 Russia; Moscow Polytechnic University 38 Bol'shaya Semenovskaya street Moscow 107023 Russia; Peoples' Friendship University of Russia 6 Miklukho-Maklaya str. Moscow 117198 Russia; Lomonosov Moscow State University Leninskie Gory Moscow 119991 Russia; Moscow Institute of Physics and Technology State University 9 Institutskiy Per., Dolgoprudny Moscow Region 141700 Russia

## Abstract

Nanostructured composite particles of nano- and submicron sizes were synthesized by a combination of sol–gel and sonochemical techniques. Their graphene content was 0.8–0.9 wt%. These layered particles consisted of graphene sheets in which zirconia nanocrystals were discretely incorporated. The synthesized powders were characterized using XRD, TEM, HRTEM, diffusion aerosol spectrometry and elemental analysis. A comparison of the compressibility modulus, limit values of linear section deformation and compressibility factor shows that the compressibility of the composite is difficult to achieve compared to that of pure zirconia, apparently, due to the low elasticity of graphene sheets.

## Abstract 2

Nanostructured composite particles of nano- and submicron sizes were synthesized by a combination of sol–gel and sonochemical techniques.

## Graphical abstract

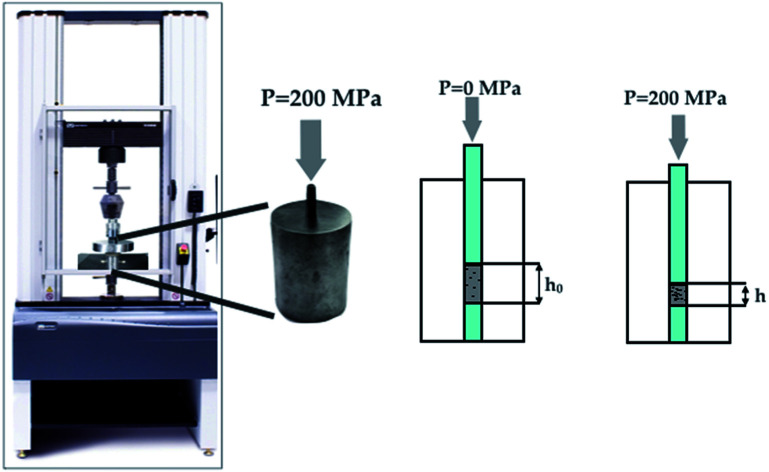

## 1 Introduction

The use of nanostructured powders allows obtaining materials with high density and electrical conductivity. They are essential for the development of small-sized high-speed electronic device production with a broad set of functions and at the same time with low energy consumption due to the presence of specific interactions within complex nanostructures and small energy losses to the environment.^1^ A synergistic effect of specific physicochemical properties was observed in composites based on graphene and transition metal oxides.^2–7^ The development of a reliable method for the synthesis of such hybrids with controlled structure and properties proved to be challenging to put into practice. One of the main problems in obtaining graphene-based composites is related to the fact that nanoparticles are not evenly distributed over graphene sheets. Homogenization processes involving graphene reinforcement are complicated due to the requirement of providing an even distribution while avoiding the introduction of oxygen-containing defects into graphene.^8^ Uniform dispersion of components in the material was obtained by dispersing graphene and the ceramic powder separately in isopropanol media and then mixing them in an ultrasonic bath under continuous mechanical stirring.^9^ Isopropyl alcohol, *N*-methylpyrrolidone and *N*,*N*-dimethylformamide are solvents that can prevent agglomeration in processes in colloidal systems.^10^

Composites consisting of graphene sheets and zirconia are promising materials for various industrial fields due to their physicochemical characteristics (excellent mechanical, electrical, thermal, optical and stable photochemical). These features of the composites are due to the presence of graphene.^11–14^ Hybrid structures consisting of nanocrystals wrapped in graphene sheets are recognized as the most effective for many relevant applications: fuel cells (catalyst carrier), solar cells, supercapacitors, electronic devices, Fischer–Tropsch synthesis catalysts, hydro processes, ammonia synthesis/decomposition, photocatalysts for environmental protection (decomposition of organic dyes and solvents), thermal protective coatings, transistors, and biocompatible matrices for immobilization of proteins.^15,16^ Developing a reliable method for the synthesis of a hybrid with controlled structure and properties was challenging to put into practice.

Only a few publications are available about graphene/zirconia composites and their applications in lithium-ion batteries, supercapacitors, or detectors of organic phosphorus agents. Compared with unoxidized graphene, graphene-oxide and reduced graphene-oxide showed a minor enhancement of fracture toughness due to the degraded mechanical strength of reduced graphene-oxide and the structural defects of graphene-oxide.^17^ It was shown that the addition of not more than 1 vol% graphene to Zr-ceramics makes it possible to obtain a composite material with increased density, hardness, and wear resistance.^18^ The inclusion of ZrO_2_ nanoparticles in graphene sheets leads to the production of promising materials for the anodes of lithium-ion batteries.^19^ This graphene/zirconia composite shows high specific capacity, high coulomb efficiency, excellent rate performance and cycling stability. Due to the sandwich-like stable structure of zirconia, its inert pillar supports graphene, which offers more active sites for Li^+^ insertion.

It was found that graphene with a nano laminated structure can effectively improve the strength and hardness of composites without reducing their plasticity,^20^ so the importance of structural design is emphasized.^21^ In ref. 22 considers the impact of graphene and its derivatives on the mechanical and electrical properties, when the graphene additive content in stabilized ZrO_2_ reaches several tens of percents.

An inexpensive and straightforward method for the synthesis of composite aerogels consisting of reduced oxidized graphene and ZrO_2_ with a mesoporous structure was proposed in the article.^23^ Epichlorohydrin and dimethylformamide were used to reduce oxidized graphene in the synthesis. Du *et al.* have mentioned the preparation, characterization, and electrochemical properties of graphene/ZrO_2_ nanocomposites and their application in the enrichment and detection of methyl parathion.^24^ However, none of the mentioned composites have been in the condensed state of aerogel, and thus have limited applications in many fields due to their quite low specific surface area.

A few years ago, some methods were developed for ZrO_2_ deposition on graphene oxide.^25–27^

Several studies^28,29^ on strengthening polymer materials with graphene oxide have been published, they clearly show the positive effect of an additive of 1–2 wt% graphene oxide on the elastic modulus and fracture toughness, which increases the strength of the composite material reinforced with graphene. The strength characteristics of the composite increased by 10–40%, the composite material successfully withstood dynamic loads at higher voltages than the initial one before graphene was dispersed in it.

In ref. 30, the graphene's influence on the structure and performance of the electrodes was reviewed; and it summarized the recent advances in graphene-containing anode electrode materials. Various methods have been developed to incorporate metal oxides into graphene-containing materials. The mechanical mixing of carbon materials with metal oxides does not produce a hybrid material with a typical structure. As an alternative, methods using liquid media (colloids), solid-phase pyrolysis, and chemical interactions are considered. The use of these methods leads to the production of decorated and mesoporous systems, core–shell particles *etc.* In ref. 31, graphene nanosheets, prepared *via* oxidative exfoliation of graphite and subsequent chemical reduction, have been selected as anode materials.

In ref. 32, a solvothermal approach to prepare stabilized cubic zirconia nanoparticles, highly reduced graphene oxide, and ZrO_2_ nanocomposite, highly reduced graphene oxide–ZrO_2_, was considered using benzyl alcohol as a solvent and stabilising ligand. Electronic interactions between ZrO_2_ and highly reduced graphene oxide were studied by comparing their electrochemical properties with those of pure ZrO_2_ and highly reduced graphene oxide using cyclic voltammetry.

In ref. 33, the effect of the graphene oxide content on the electrochemical properties of the composite was investigated, and it was found that an increase in the ZrO_2_/graphene oxide mass ratio above 1/2 leads to a decrease in the specific capacitance of the composite. It was suggested that the improvement in the electrochemical properties is due to the nanoscale components and the high electronic conductivity of graphene oxide. Both of these factors contribute to the enhanced charge-transfer-reaction pseudocapacitance of the nanocomposite owing to rapid and reversible redox reactions at its surface.

Despite a large number of publications on ZrO_2_–graphene oxide composites, there is still no reliable and technologically promising method for the production of composites based on oxygen-free graphene and ZrO_2_. The difficulties in synthesising an electrode material with a stable structure have not yet been overcome. A challenge associated with the problem of obtaining high-quality graphene, free of oxygen-containing functional groups and with a low agglomeration degree, also remains unresolved. We proposed a method for the synthesis of composite nanostructured powder, which combines sol–gel and sonochemical methods. This approach ensures an uniform distribution of both components, graphene sheets and zirconia nanocrystals, in the composite due to the fact that the mixing of the components is carried out at the molecular level.

We have developed a method for producing hybrid nanostructures based on oxygen-free graphene and metal oxides with a nanocrystal size of 3 nm. The uniqueness of this method lies in the combination of sol–gel synthesis and ultrasonic exfoliation of graphene sheets from the surface of synthetic graphite in an organo–inorganic medium and in the synthesis of sol and in the exfoliation of graphene in the same organic amine.^34–36^ This method ensures an uniform distribution of components in the nanopowders, almost every nanocrystal of metal oxide is wrapped with a graphene sheet. Such nanostructured powders as raw products are very interesting and promising for the synthesis of new types of ceramics with improved physicochemical properties. At the same time, the rheological properties of such powders need to be specifically investigated. It was shown that the presence of sheets of 0.05 wt% oxidized graphene in cement leads to an increase in compressive strength and flexural strength of the material by 15–33 and 41–59%, respectively.^37–39^ However, it is not known yet if there is any study of nanocarbon species inclusion effect on the rheological properties of nano- and submicron powders. We carry out comparative research of the nanopowders of a graphene-containing composite and pure zirconia obtained from the same sol as a composite. Therefore, we combined physicochemical characterization and the rheological test results of the powders.

## 2 Experimental

### 2.1. Synthesis of nanostructures

For the synthesis of hybrid nanostructures, the as-prepared Zr-containing sol and a graphene suspension after sedimentation and decanting were used.

The Zr-containing sol was synthesized using zirconyl nitrate dehydrate, ZrO(NO_3_)_2_·2H_2_O (TU 6-09-140676, ChimMed, Russia, purity is 99.5%). The concentration of the salt stock solution in deionized water was 0.05 M. Acetylacetone (AcAcH, GOST 10259-78, ChimMed, Russia, purity is 99.5%) and *N*,*N*-dimethyloctylamine (DMOA, Acros Organics, CAS: 7378-99-6, purity is 97%) were used for the complexation and stabilization of sol. The weight ratio of AcAcH/DMOA was 1.5. In the synthesis of Zr-containing sol, the molar ratios of DMOA/Zr and AcAcH/Zr were 2.0 and 4.8, respectively. For the formation of a mesophase, a water–alcohol mixture was used with a water/ethanol volume ratio of 2. The sol was synthesized in a flask with a reflux condenser on a magnetic stirrer at a temperature of 80–90 °C.

The graphene sheet suspension was obtained by ultrasonic exfoliation of the graphite surface in the aqua-DMOA emulsion with pH 3. The initial graphite powder (Processing Science, Russia) had a particle size of 600–800 microns and a purity of 99.99%, while the impurities presented as sulfur (less than 10 ppm) and chloride ions (10 ppm). The obtained graphene sheets were passed to the acidic substrate, where their stabilization took place through the fixation at the aqua-DMOA boundary. Then the obtained graphene suspension was used to synthesize hybrid nanoparticles after sedimentation and decanting.

For the obtaining of a mixed colloid, the as-prepared Zr-containing sol and graphene suspension were mixed under stirring (500 rpm) and heating to 60–65 °C on a magnetic stirrer for 20–25 minutes. Then it was evaporated at 95–98 °C and stirred on a magnetic stirrer (500 rpm) until a gel was formed, which was transferred to a porcelain cup and placed in an oven. The subsequent heat treatment was carried out in air at 500 °C for 1 h, resulting in the formation of a fine light beige powder.

### 2.2. Characterization of phase composition and morphology

The phase composition of graphene–zirconia and pure zirconia powders was investigated by XRD (DRON-3M, Russia, with the use of CuKα radiation) and using the JCPDS card index and interpretation software for XRD, PHAN and PHAN%.^40,41^ The morphology of the obtained powders was investigated by transmission electron microscopy (TEM) with use of a LEO-912 AB OMEGA instrument at 100 kV. The powder particle size distribution was investigated using an automated diffusion aerosol spectrometer, Model 2702, Aeronanotech (DAS). The weighed portion of the studied powder was 0.001 g; the ultrasonic treatment duration of the sample was 3 min. The relative measurement error was 5%. The HRTEM data and the EDS spectra were obtained on a JEM 2010 instrument (JEOL Ltd.) equipped with an energy dispersive spectrometer (EDS; Inca, Oxford Instruments). The thickness and the number of layers in the sheets were calculated based on 5–10 measurements on each of 5–6 TEM and HRTEM microphotos for each composite sample. The carbon content of the powders was determined using an elemental analyzer (Carbon Sulfur LECO SC-400 Analyzer). The bulk density of the synthesized powders after shaking was determined by the Scott method in accordance with international standard ASTM B329-18 (ref. 42) using the formula: *ρ* = *m*/25.

### 2.3. Rheological tests of the synthesized powders

**Fig. 1:**
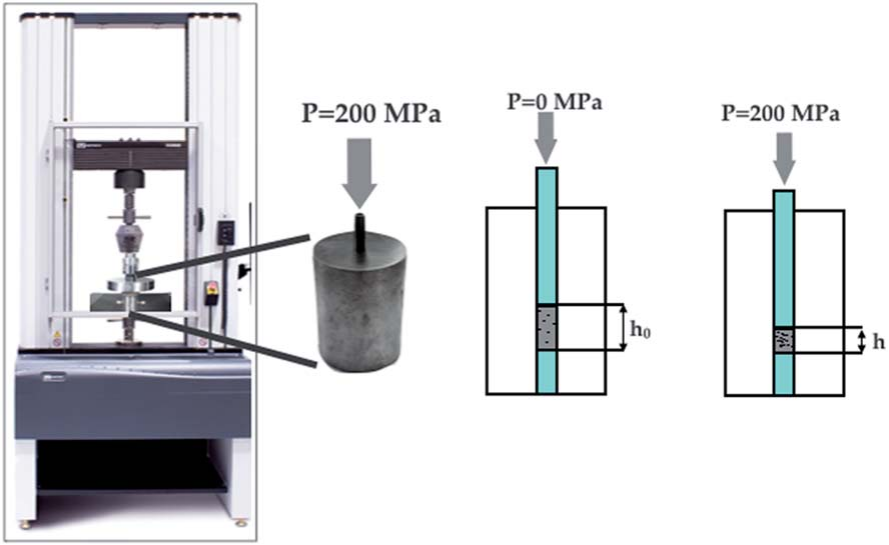
The schematic loading diagram of the rheological test.

The rheological properties were tested on a mechanical testing machine (Instron 5581, Great Britain). The plunger velocity was 1 mm min^−1^. A sample of the test sample weighing 0.15 g was placed in a steel mould. At least 5 measurements were carried out for each sample, then the values were averaged (the average value of the standard deviation of deformation in the entire interval of the experiment was *σ*_1_ = 0.0744 and *σ*_2_ = 0.0476 for graphene/ZrO_2_ and pure ZrO_2_, respectively), and calculations were carried out using the formulas below. The average value of the standard deviation of deformation in the entire interval of the experiment was *σ*_1_ = 0.0744 and *σ*_2_ = 0.0476 for graphene–zirconia composite and pure zirconia powders, respectively. The schematic loading diagram is presented in Fig. 1.

The deformation was calculated by the equation:

1 *ε* = Δ*h*/*h*_0_,

where Δ*h* is the change in the height of the bulk layer over time; *h*_0_ is the initial height of the bulk layer.

The compressibility modulus is numerically equal to the tangent of the linear section slope angle of the diagram strain against deformation, and is calculated by the equation:

2 *G* = Δ*p*′/ε*,

where Δ*p*′ is the limiting change in the linear section pressure value; ε* is the final value of the linear section deformation. The linear section was selected based on the condition of the deviation of the experimental data from the linear curve by more than 2%.

The compressibility coefficient (*k*_c_) characterises the reversible decrease in the sample height (volume) under the pressure conditions and is quantitatively determined by the equation:3
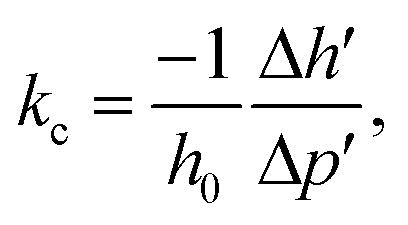
where Δ*h*′ and Δ*p*′ are the limiting values of the height and the linear section pressure variation and *h*_0_ is the initial height of the backfill.

## 3 Results and discussion

**Fig. 2:**
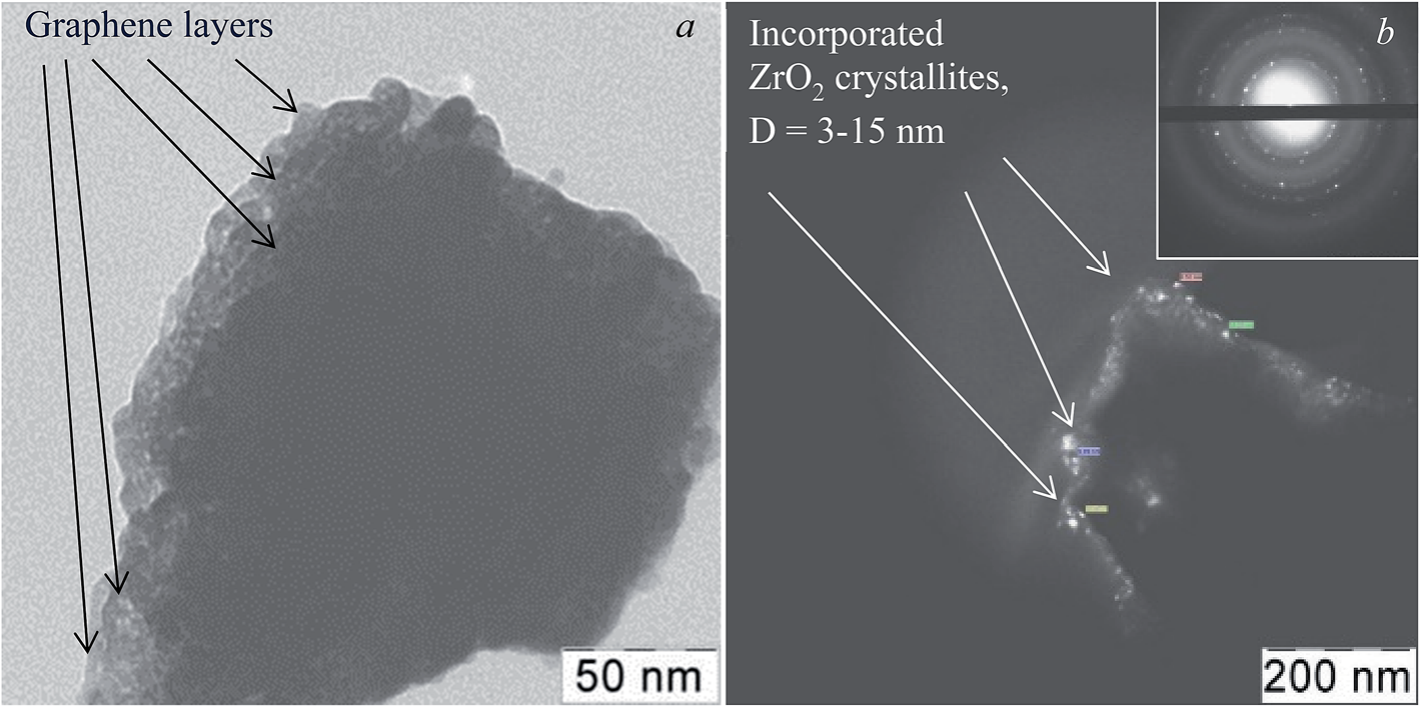
TEM images of the graphene–zirconia composite powder: in bright field (a), in the dark-field image (b) and electron diffraction (inset).

A feature of the proposed synthesis method is the use of a combination of sol–gel and sonochemical techniques: a sol-intermediate was used to synthesize a composite. In this case, sol–gel transition and subsequent zirconia crystallization take place in the presence of semi-transparent graphene sheets in the liquid substrate, which is a three-phase colloid. As a result, a composite with the structure of van der Waals system was formed, as shown earlier.^36^ According to elemental analysis, in the obtained composite graphene–zirconia powders, the carbon content was 0.80–0.90 (±0.02) wt%. It was incorporated into the composite structure as nanometer thickness graphene sheets (Fig. 2a), in which, the absence of oxygen was proved by the EELS analysis earlier.^34^

TEM image in Fig. 2a shows that the composite powder consists of layered agglomerates with linear dimensions of up to several hundred nanometers. The zirconia crystallites are discretely incorporated into graphene layers almost uniformly. The dark-field image in Fig. 2b shows that discrete inclusions have sizes of 3 to 15 nm, despite the fact that, in general, graphene prevents the intensive particle agglomeration both of the initial sol and crystallites. Possibly a small amount of agglomerated particles is observed. They were formed due to the high surface energy of the nanoparticles. The electron diffraction pattern is shown in the inset of Fig. 2; it corresponds to a lot of differently oriented zirconia crystallites that belong to several modifications.

**Fig. 3:**
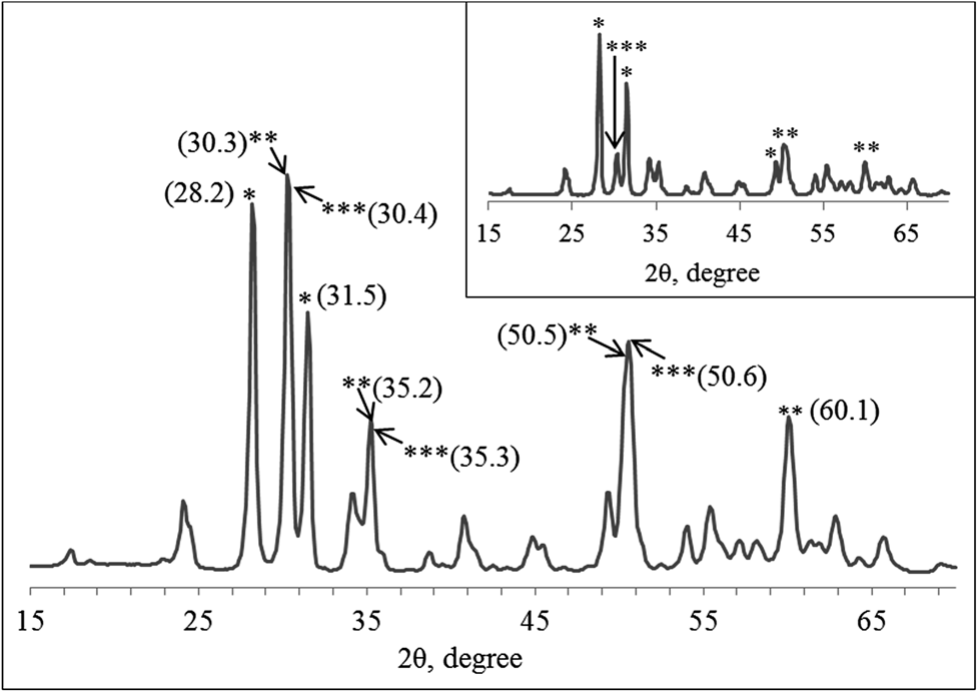
XRD pattern of the graphene–zirconia composite and pure zirconia nanopowder obtained from the same sol (inset).

According to the XRD data (Fig. 3), the phase composition of the hybrid nanostructured powder is heterogeneous, since three groups of Bragg's reflections are observed on its diffractogram. The raflections at 2*θ c.a.* 28.2 (111) and 31.5 (111) correspond to monoclinic ZrO_2_ (JCPDS card no. 78-078-1807); the reflections at 2*θ c.a.* 30.3 (111), 35.2 (200), 50.5 (220), and 60.1 (311) correspond to tetragonal ZrO_2_ (JCPDS card no. 01-080-2155); the reflections at 2*θ c.a.* 30.4 (111), 35.3 (200), and 50.6 (220) correspond to tetragonal ZrO_1.99_ (PCPDF card # 802155). The XRD pattern shows that in the composite, zirconia is present as monoclinic and tetragonal crystallographic ZrO_2_ modifications, and in the nanocrystalline non-stoichiometric ZrO_1.99_ phase. The same set of peaks can be seen in the diffractogram of the pure zirconia nanopowder obtained from the same Zr-containing as-synthesized sol. However, the peak ratio is different from that of the composite (Fig. 3, inset).

Table 1 shows the contents of various zirconia crystallite modifications and the morphological and structural parameters calculated from the XRD data for the graphene–zirconia composite and pure zirconia nanostructured powders, which were synthesized from the same sol. In the composite, the content of monoclinic zirconia modification was reduced by 28% compared to the pure zirconia powder, while the content of tetragonal non-stoichiometric ZrO_1.99_ increased 3.6 times. In the composite, the dispersion of monoclinic zirconia and tetragonal non-stoichiometric ZrO_1.99_ remains, however, the dispersion of the tetragonal modification increases by more than 2 times. In the composite, the lattice parameters of the tetragonal ZrO_2_ also differ from those of the pure zirconia powder.

**Table 1:** Phase composition, dispersion, and lattice parameters of zirconia in the graphene–zirconia composite and pure zirconia nanostructured powders (according to the XRD data). D* – the average crystallite size

Zirconia modification	Graphene–zirconia				Zirconia (pure)			
	Content, wt%	D*, nm	Lattice parameters	Å	Content, wt%	D*, nm	Lattice parameters	Å
Monoclinic ZrO_2_	63	8	*a*	5.15	87	8	*a*	5.15
			*b*	5.19			*b*	5.19
			*c*	5.33			*c*	5.32
Tetragonal ZrO_2_	8	6	*a*	3.62	5	14	*a*	3.60
			*c*	5.16			*c*	5.13
Tetragonal ZrO_1.99_	29	8	*a*	9.56	8	8	*a*	9.54
			*c*	17.58			*c*	17.59

**Fig. 4:**
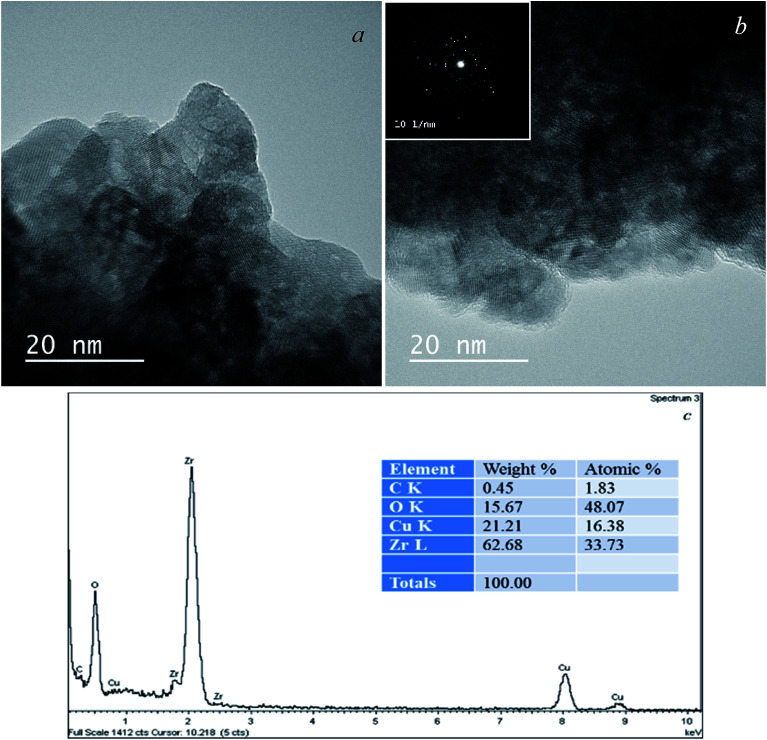
HRTEM images (a and b), the EDS spectrum with elemental distribution (c) of the graphene–zirconia composite and electron diffraction (inset).

According to the HRTEM data (Fig. 4a and b), the layered composite agglomerates consist of graphene sheets with a thickness of not more than 1 nm, *i.e.* no more than 3 layers. Electron diffraction shows the presence of mainly 1-2-layer sheets of graphene and polycrystalline zirconia (inset). The sizes of zirconia crystallites incorporated in graphene are mainly 3–10 nm and do not exceed 15 nm, which corresponds to the TEM and XRD data (Fig. 2, 3 and Table 1).

Fig. 4c shows the EDS spectrum of the composite, it indicates the presence of elements C, O, and Zr in the sample. Сarbon comes from graphene, while Zr and O are from zirconia. The Zr/O atomic ratio is close to 1.5, which indicates a large fraction of oxygen vacancies in the zirconia crystal lattice. The Cu-related peaks in the spectrum originate from the copper grids.

**Fig. 5:**
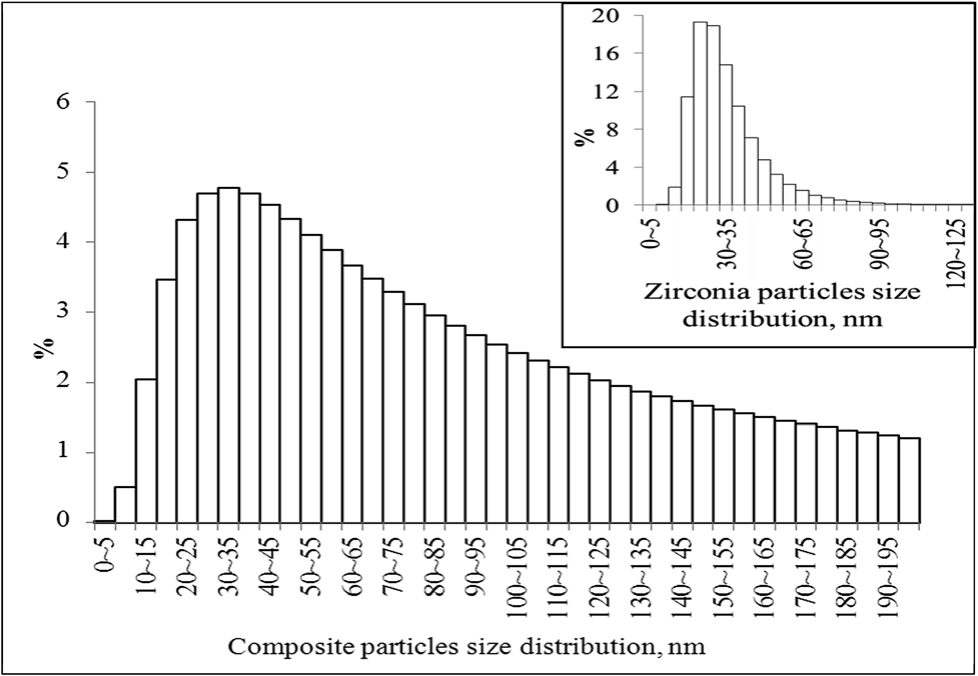
Particle size distribution of the graphene–zirconia composite powder and the pure zirconia nanopowder (inset).

Fig. 5 shows a wide size distribution of composite particles: species with sizes less than 100 nm are more than 60%, more than 90% consist of the particles with sizes up to 200 nm and less than 10% are larger particles. The TEM data for such particles were presented above in Fig. 2. The inset of Fig. 5 shows the zirconia particle size distribution in the pure nanopowder, and the calculated average particle agglomerate size was 28 nm.

Probably, the powder of pure zirconia consisted of agglomerates that included no more than 4 crystallites. Apparently, the sizes of composite particles are determined by the linear sizes of graphene sheets in the presence of which crystallization of zirconia occurs. The Scott density (ASTM B329-18) of the synthesized powders was 1.30 (±0.05) and 1.50 (±0.06) g cm^−3^ for pure zirconia and composite powders, respectively.

**Fig. 6:**
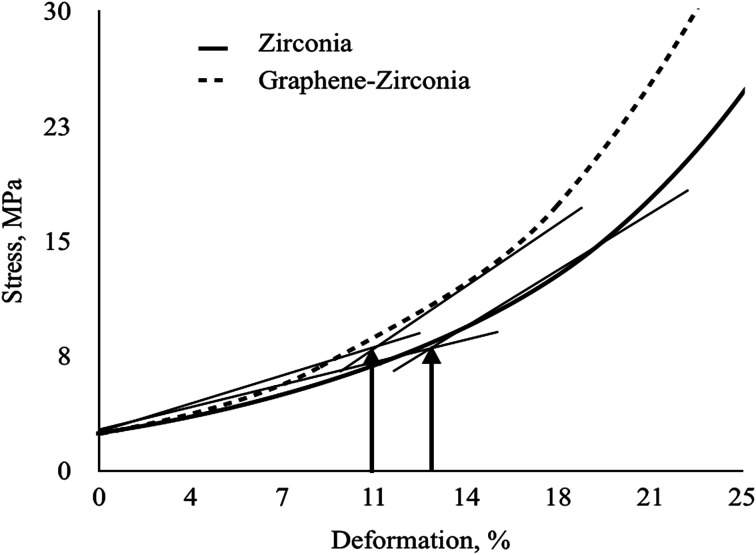
Compaction curves on the “deformation–pressure” plane of graphene–zirconia and pure zirconia nanostructured powders.

Since the developed powdery composites are intended for ceramics, a comparative study of the compressibility of the nanostructured graphene–zirconia composite and pure zirconia powders was carried out. Fig. 6 shows the typical “deformation–pressure” curves of the composite and pure zirconia powders constructed on the basis of the results of 5–7 independent numerical experiments and kinetic studies of the pressure dependence on exposure time. The ultimate strain values corresponding to deviations from the linear compression of the powders are also noted.

As can be seen, at low pressures, the curves increased monotonically linearly at the initial stage, which corresponds to elastic deformation, when the stress–strain relation obeys Hooke's law. Then the curves become bent due to the transition of the systems to the plastic deformation stage due to the generation of dislocations.

The tangent angle of the linear portions of both curves is equal to the compressibility modulus; therefore, the compressibility modulus of the hybrid nanopowders is much higher than that of pure zirconia. A comparison of the curves shows that the composite goes into the region of plastic deformation at a lower pressure than the pure zirconia nanopowder. It can also be observed that in order to achieve the same degree of deformation, higher pressure is required for the pure ZrO_2_ nanopowder.

Table 2 shows the calculated parameters of compressibility of the graphene–zirconia composite and pure zirconia nanostructured powders. As can be seen, the compressibility modulus of the composite is one and a half times higher relative to that of the pure zirconia nanopowder. The limit strain value of the composite decreased by 8 percent relative to that of pure nano-zirconia. Wherein, the compressibility factor of the composite also decreased by 42% relative to that of pure nano-zirconia.

**Table 2:** Calculated parameters of compressibility of the graphene–zirconia composite and pure zirconia nanostructured powders (reliability is 98%)

Powder	Compressibility modulus, *G*, MPa	Limit values of linear section deformation, E*, %	Compressibility factor, ×10^5^, Pa^−1^
Graphene–zirconia	44.8	11.4	1.25
Zirconia	30.0	12.4	2.14

## 4 Conclusions

A composite based on zirconia and oxygen-free graphene was synthesized by a combination of sol–gel and sonochemical techniques. During its synthesis, crystallization was carried out from a liquid substrate in the presence of the as-prepared Zr-containing sol. As a result, layered composite particles of nano- and submicron sizes were formed. In the composite, the zirconia crystallites with sizes less than 15 nm were incorporated into graphene sheets of 2–3 nm thickness. In the composite and in the pure zirconia nanopowder, the dispersion, phase composition, and lattice parameters of zirconia are different, despite the fact that the pure zirconia was obtained from the same sol as the composite. Apparently, graphene sheets have a significant impact on the zirconia crystallization, despite the fact that the graphene content in the composite does not exceed 1 wt%. The structural and phase differences of the powders lead to a difference in their density and compressibility. A comparison of the compressibility modulus, limit values of linear section deformation, and compressibility factor shows that the compressibility of the composite is difficult to achieve compared to that of pure zirconia due to the low elasticity of graphene sheets. It shows up in the first and second stages of pressing, *i.e.* in the region of elastic–plastic deformation, when optimization of the particle distribution in the sample volume and then compaction due to accommodation and deformation of the powder particles occur, respectively. As a result, it was found that the introduction of even 1 wt% oxygen-free graphene into the zirconia nanopowder significantly affects its rheological properties, reducing the compressibility factor up to two times due to the elasticity of the incorporated graphene sheets.

## Conflicts of interest

There are no conflicts to declare.

## Supplementary Material
